# Severe Manifestations of Human Metapneumovirus With Co-infections: A Case Series and Literature Review

**DOI:** 10.7759/cureus.99262

**Published:** 2025-12-15

**Authors:** Aradhana, Viveka Mishra, Amit R Rup, Nirmal K Mohakud

**Affiliations:** 1 Pediatrics, Kalinga Institute of Medical Sciences, Bhubaneswar, IND; 2 Pediatric Medicine, Kalinga Institute of Medical Sciences, Bhubaneswar, IND

**Keywords:** co-infections, extrapulmonary complications, human metapneumovirus, molecular diagnostics, pediatric critical care

## Abstract

Human metapneumovirus (hMPV) causes severe pediatric respiratory infections, often with co-infections. This report details three immunocompetent children with hMPV co-infected with adenovirus, SARS-CoV-2, *Streptococcus pneumoniae*, and *Acinetobacter baumannii*, causing pneumonia, respiratory failure, and systemic complications (hypertension, cardiac arrest, neurological sequelae), requiring prolonged mechanical ventilation and broad-spectrum antimicrobials in the ICU. Diagnostic delays from overlapping features required BioFire® (BioFire Diagnostics, Salt Lake City, UT) testing. Studies link hMPV co-infections to heightened severity, prolonged hospital stays, and ICU admissions, yet guidelines are lacking. Rapid molecular diagnostics guide targeted therapy and antimicrobial stewardship. Extrapulmonary manifestations (dystonia, optic atrophy) reflect hMPV’s systemic effects. These three cases highlight clinical vigilance and urgent research into antivirals/vaccines for vulnerable populations.

## Introduction

Human metapneumovirus (hMPV) is a single-stranded, negative-sense RNA virus, a member of the Pneumoviridae family. Structurally, it is an enveloped virus with a helical nucleocapsid, featuring surface glycoproteins (F, G, and SH) that mediate host cell entry and immune evasion [1]. hMPV primarily infects respiratory epithelial cells and is a significant cause of acute respiratory tract infections (ARTIs) across all age groups, particularly in young children, immunocompromised individuals, and the elderly [1,2]. Indian studies found an average hMPV prevalence of 5% among children with ARIs, peaking at 7% in the northeast region and 6% in children under five [3]. It accounts for 5-15% of ARIs in hospitalized children worldwide, with higher rates in low- and middle-income countries (LMICs) [4]. hMPV exhibits seasonal patterns, peaking in late winter and spring, often overlapping with respiratory syncytial virus (RSV) and influenza, complicating clinical differentiation [1,5]. While hMPV typically manifests as mild upper respiratory illness, severe lower respiratory tract infections (LRTIs), including bronchiolitis, pneumonia, and acute respiratory distress syndrome (ARDS), are well-documented in high-risk populations [6]. Immunocompromised patients, such as hematopoietic stem cell transplant recipients, face heightened risks of progressive pneumonia and mortality, with limited therapeutic options beyond supportive care [7].

Studies report hMPV as the etiology of 4-13% of adult pneumonia cases requiring hospitalization, with outcomes comparable to influenza and RSV [8,9]. The Pneumonia Etiology Research for Child Health (PERCH) study found hMPV in 6.9% of severe pneumonia cases in children under five, making it the second most common viral pathogen after RSV [4]. However, its role in pediatric critical illness, particularly in the context of co-infections, remains undercharacterized. Co-infections with bacterial or viral pathogens are increasingly recognized as drivers of severe disease, yet data on their clinical impact are limited [4]. For instance, hMPV-bacterial co-infections exacerbate inflammation and prolong hospitalization, with *Streptococcus pneumoniae* and *Klebsiella pneumoniae *identified as common co-pathogens [10,11]. Viral co-infections, such as adenovirus or SARS-CoV-2, may further complicate immune responses and therapeutic management [12]. In such scenarios, the delayed diagnosis may lead to an increase in disease severity and catastrophic events.

Current literature emphasizes the diagnostic challenge posed by hMPV’s nonspecific presentation, which often mirrors bacterial pneumonia, leading to delayed diagnosis and unnecessary antibiotic use [13]. Furthermore, extrapulmonary complications, including myocarditis, seizures, and neurological sequelae, are rare but increasingly reported, underscoring the virus’s systemic potential [14,15]. This case series aims to contribute to the evolving narrative of hMPV’s clinical spectrum by highlighting severe pediatric manifestations compounded by co-infections. Our findings underscore the importance of early molecular diagnostics to guide targeted therapy, reduce antimicrobial overuse, and improve outcomes.

## Case presentation

Case 1

**Figure 1:**
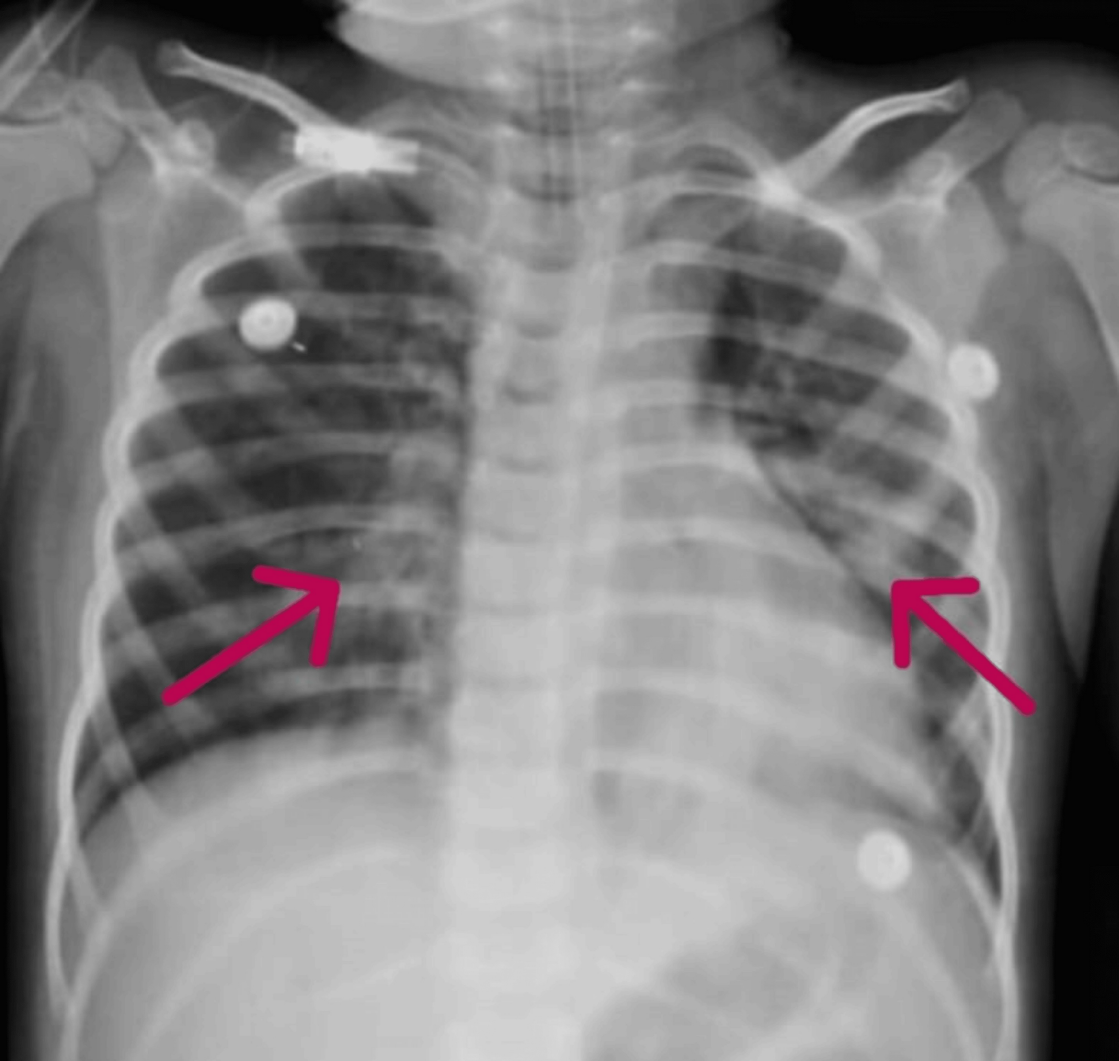
Chest X-ray (AP view) showing non-homogenous patchy opacities in bilateral lung fields, more prominent in the lower zones

A 2.5-year-old male with global developmental delay (post-hypoxic-ischemic encephalopathy (HIE) at birth) and seizure disorder presented to the emergency department with a two-day history of productive cough, high-grade fever (102°F), and an episode of abnormal body movements. On admission, he exhibited severe respiratory distress (nasal flaring, head bobbing, subcostal retractions) and was stabilized with high-flow oxygen, levosalbutamol nebulization, and paracetamol before transfer to the pediatric intensive care unit (PICU). Initial investigations revealed marked elevation of sepsis markers (C-reactive protein (CRP): 134 mg/dL (normal <5 mg/dL), procalcitonin: 16.2 ng/mL (normal <0.5 ng/mL); Table 1). Chest X-ray demonstrated bilateral non-homogeneous patchy opacities consistent with pneumonia (Figure 1).

**Table 1:** Key laboratory parameters of Case 1 MP-ICT: malaria parasite immunochromatographic test; OD: optical density; Dengue IgM/NS1: a diagnostic blood test for the dengue virus that detects two components: IgM (immunoglobulin M), which indicates a recent or current infection, and NS1 (non-structural protein 1)

Parameter	Day 1	Day 3	Reference Range
C-Reactive Protein (mg/dL)	134.7	55.8	<5
Procalcitonin (ng/mL)	16.2	0.799	<0.5
Hemoglobin (g/dL)	10.2	7.3	11.5–14.5
Total Leukocyte Count (×10³/µL)	8.57	6.73	6.0–17.5
Sodium (mEq/L)	134	133	135–145
BioFire®) (BioFire Diagnostics, LLC. (2022). *BioFire FilmArray Respiratory Panel 2.1 (RP2.1))*	Human Metapneumo-Virus, Adenovirus		
Malaria Parasite-ICT		Negative	
Dengue IgM/NS1		Negative	
Scrub Typhus IgM (OD)		0.05	>0.5

Differential Diagnoses and Investigations

Bacterial pneumonia with sepsis: Elevated CRP and procalcitonin prompted empiric broad-spectrum antibiotics (piperacillin-tazobactam, later escalated to linezolid and meropenem). Antibiotic therapy was escalated due to clinical deterioration alongside markedly elevated procalcitonin (16.2 ng/mL), strongly suggesting bacterial co-infection. Blood, urine, and endotracheal cultures were performed but yielded no significant pathogens.

Atypical or viral pneumonia: Persistent fever and worsening respiratory distress (requiring intubation on day three) led to adjunctive azithromycin and oseltamivir. A BioFire® respiratory panel later confirmed co-infection with adenovirus and hMPV. The patient's significant respiratory compromise is attributed to the dual viral infection with hMPV and adenovirus, with elevated biomarkers guiding the suspicion of an undetected bacterial component.

**Figure 2:**
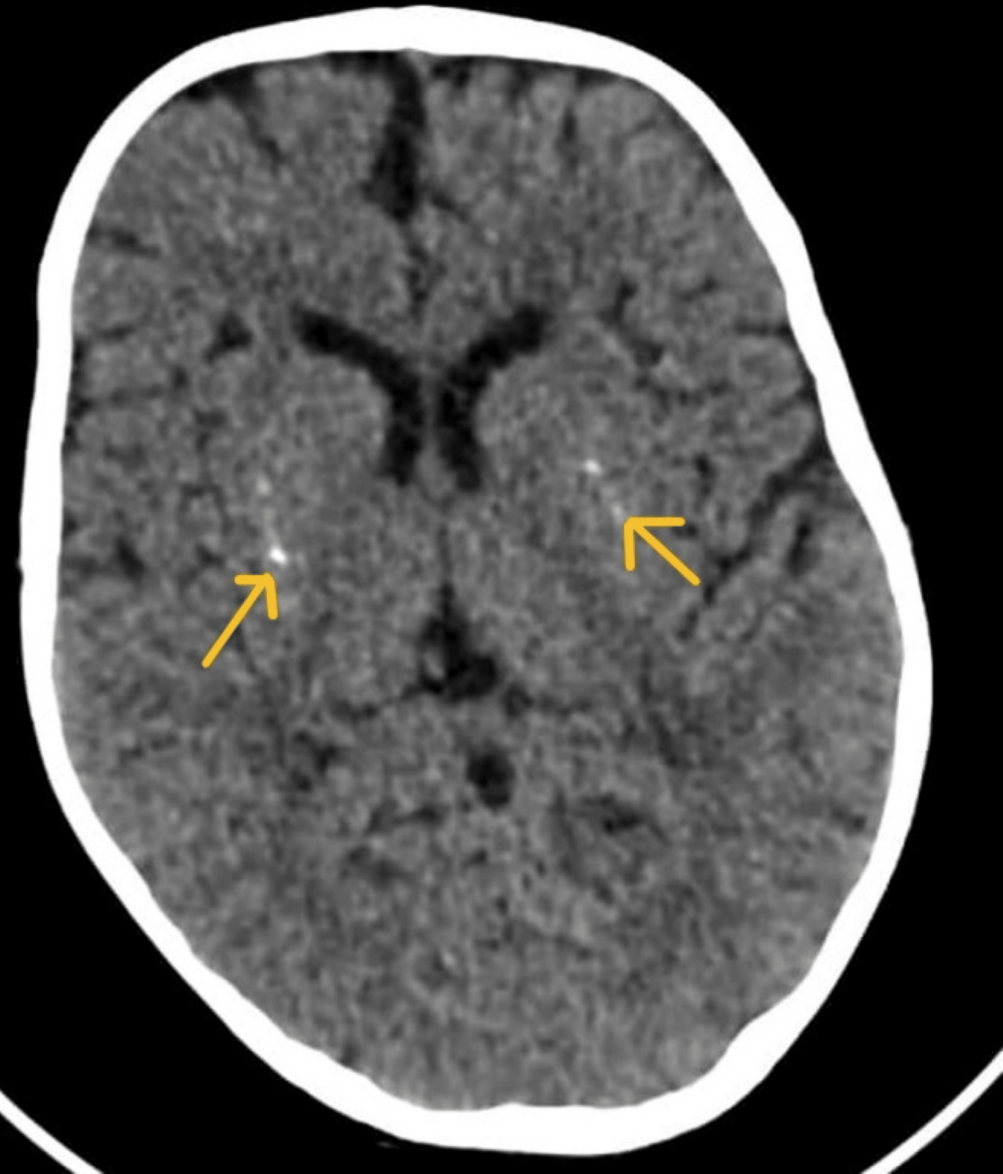
Non-contrast axial computed tomography (CT) of the brain demonstrating symmetrical, bilateral calcification within the lentiform nuclei (arrows)

Neurological evaluation was initiated due to seizures and dystonia. Contrast-enhanced computed tomography (CECT) of the brain revealed bilateral lentiform nucleus calcifications (Figure 2). Ophthalmology review identified partial optic atrophy, likely related to sequelae of post-hypoxic-ischemic injury at birth. 

Clinical Course

The child required prolonged ventilatory support and inotropes. Despite escalating antibiotics (colistin added on day 10), it took two weeks for the fever to resolve. Post-extubation (day 13), following this severe infection, he acutely developed new-onset dystonia, a significant change from his baseline. This acute deterioration was managed with pacitane, baclofen, and piracetam and is considered a sequela of the critical illness and physiological stress, not a feature of his pre-existing condition. By day 16, he was stabilized on room air and discharged with multidisciplinary follow-up at the neurodevelopmental clinic.

Outcome

The child was discharged on day 16 with residual partial optic atrophy and dystonia, requiring ongoing physiotherapy and neurorehabilitation. This case underscores the severity of hMPV-adenovirus co-infections in children with comorbidities and highlights the critical role of rapid molecular diagnostics (e.g., BioFire®) to guide therapy and reduce antibiotic overuse.

Case 2

**Figure 3:**
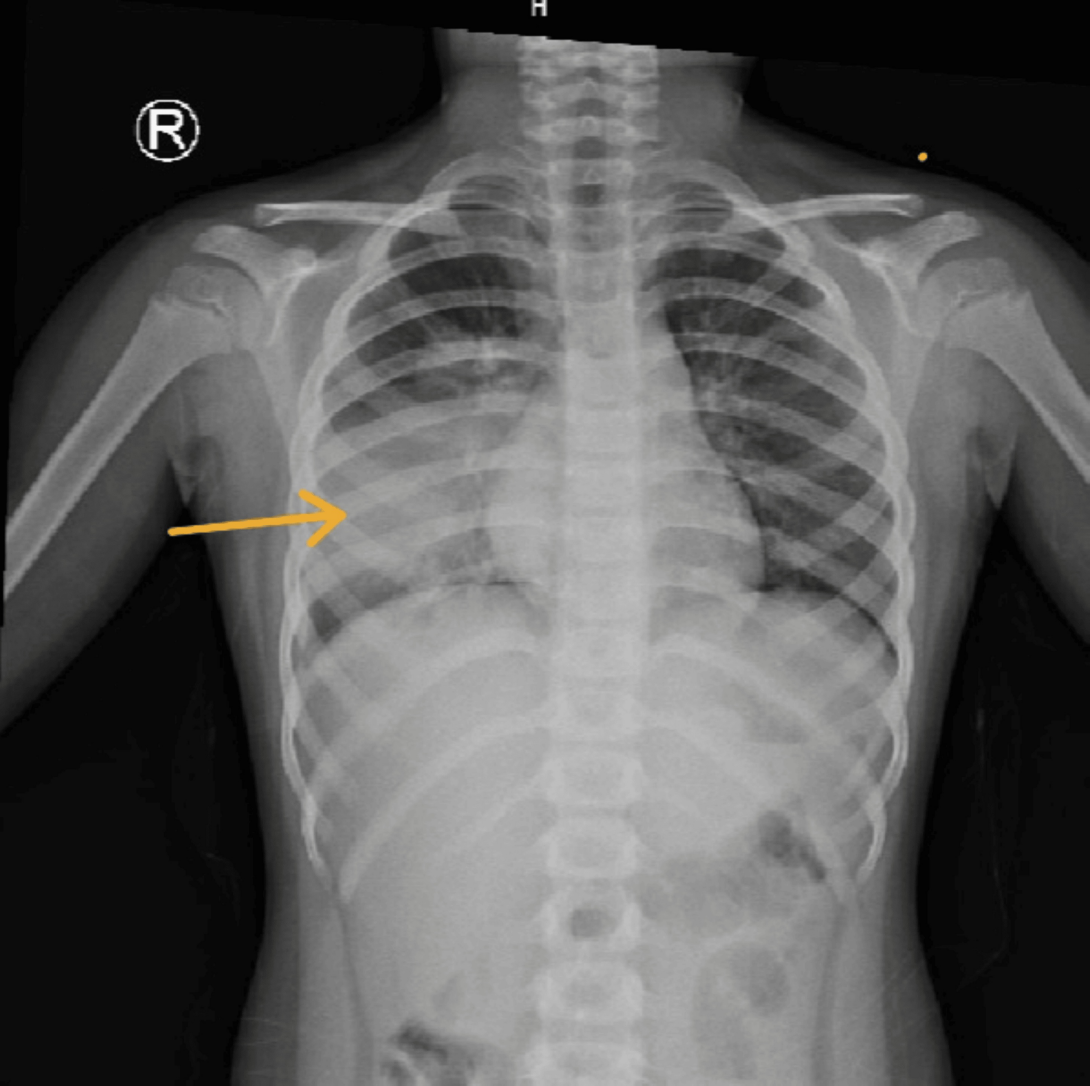
X-ray chest PA view showing homogenous opacities in the right lower and middle lobes (arrow)

A five-year-old male child presented to the emergency department with a two-day history of high-grade fever (101°F), cough, and vomiting. On examination, he exhibited bilateral crepitations and decreased breath sounds over the right axillary area. Initial management included intravenous fluids, paracetamol, levalbuterol nebulization, and ceftriaxone (100 mg/kg/day, every 12 hours). Laboratory investigations revealed markedly elevated sepsis markers: procalcitonin of 99 ng/mL (normal: <0.5 ng/mL) and CRP of 241 mg/L (normal: 0-5 mg/L) (Table 2). Chest X-ray demonstrated right lower lobe consolidation (Figure 3).

**Table 2:** Key laboratory and imaging findings of Case 2 Dengue IgM/NS1: immunoglobulin M/non-structural protein 1; MP-ICT test: malaria parasite immunochromatographic test; CBNAAT: cartridge-based nucleic acid amplification test, a rapid molecular diagnostic test that quickly diagnoses tuberculosis; OD: Optical density

Parameter	Admission Value	Reference Range
Procalcitonin (ng/mL)	99	<0.5
C-Reactive Protein (mg/L)	241	0–5
Chest X-ray	Right lower lobe opacity	
Malaria Parasite-ICT		Negative
Dengue IgM/NS1		Negative
Scrub Typhus IgM (OD)		0.28
CBNAAT testing of Sputum		Negative

Clinical Course

On day two, the child developed worsening respiratory distress, necessitating transfer to the PICU. He was initiated on heated humidified high-flow nasal cannula (HHHFNC) oxygen therapy, and antibiotics were escalated to vancomycin (10 mg/kg every 12 hours) and azithromycin syrup (15 mg/kg/day). A BioFire® respiratory panel from nasopharyngeal swabs identified co-infection with *Streptococcus pneumoniae*, hMPV, and SARS-CoV-2. Endotracheal (ET) aspirate culture grew *Acinetobacter baumannii*, sensitive to the ongoing antibiotic regimen. Tuberculosis was ruled out via the cartridge-based nucleic acid amplification test (CBNAAT).

Repeat sepsis markers on day three showed declining trends (procalcitonin: 45 ng/mL; CRP: 120 mg/L), correlating with clinical improvement. By day three, the child was weaned to low-flow nasal prongs and transitioned to room air by day four, enabling transfer back to the pediatric ward. He received chest physiotherapy to enhance lung function and was advised to undergo spirometry for further respiratory rehabilitation.

Outcome

The child responded well to antimicrobial therapy with vancomycin and azithromycin and supportive care, with resolution of respiratory distress and fever. He was discharged on day seven with recommendations for continued physiotherapy and outpatient spirometry. This case highlights the diagnostic and therapeutic challenges posed by polymicrobial co-infections (viral-bacterial) in pediatric pneumonia and underscores the utility of rapid molecular diagnostics in guiding management.

Case 3

A three-year-old male child, known case of developmental delay with HIE sequelae and seizure disorder, presented with fever and cough for four days and increasing difficulty in breathing for one day. On admission, he was in severe respiratory distress characterized by tachypnea, tachycardia, and intercostal and subcostal retractions. He was also in shock.

The child was admitted to the PICU and started on oxygen, intravenous (IV) fluids, inotropes, and IV antibiotics (ceftriaxone and doxycycline). His anti-epileptic medications (levetiracetam, sodium valproate, clobazam) were continued.

Chest radiography was suggestive of right upper lobe pneumonia. In view of no clinical improvement, antibiotics were escalated to IV vancomycin and meropenem. Despite treatment, his respiratory distress worsened with deterioration in sensorium, necessitating mechanical ventilation. He subsequently required multiple inotropic supports.

Workup and Management

The tropical fever panel (malaria parasite ICT, typhoid IgM, dengue NS1/IgM, scrub typhus IgM, and PCR) was negative, so doxycycline was discontinued. All relevant investigations are provided in Table 3.

**Table 3:** Serial investigations of Case 3 TLC: total leukocyte count

Parameter	Day 1	Day 3	Day 5	Day 7	Day 9	Day 12	Day 15
TLC (cells/cmm)	26,010	29,050	18,190	15,430	13,300	15,990	–
Differential count (%N/%L)	69/26	85/11	69/28	60/33	46/40	43/51	–
Platelet count (/cmm)	173,000	100,000	100,000	75,000	219,000	567,000	–
Hemoglobin (g/dL)	8.9	8.9	8.5	8.5	7.8	9.2	–
C-Reactive Protein (mg/L)	237	–	–	–	12	–	–
Procalcitonin (µg/L)	49	–	–	–	1	–	–
Serum urea (mg/dL)	40	56	118	167	76	65	47
Serum creatinine (mg/dL)	0.4	0.5	1.4	1.7	0.8	0.6	0.5
Serum Na (mmol/L)	139	142	138	142	137	140.1	135
Serum K (mmol/L)	4.2	4.5	4.2	5.2	2.8	3.7	4.3
Serum albumin (g/dL)	2.2	1.9	2.3	–	–	3.5	–

Renal function tests revealed worsening renal parameters; hence, vancomycin was replaced with teicoplanin. With persistent sepsis features and elevated inflammatory markers, IV tigecycline was added. A multiplex pneumonia panel was positive for hMPV, *Haemophilus influenzae *type b, and adenovirus. Blood and urine cultures were sterile.

**Figure 4:**
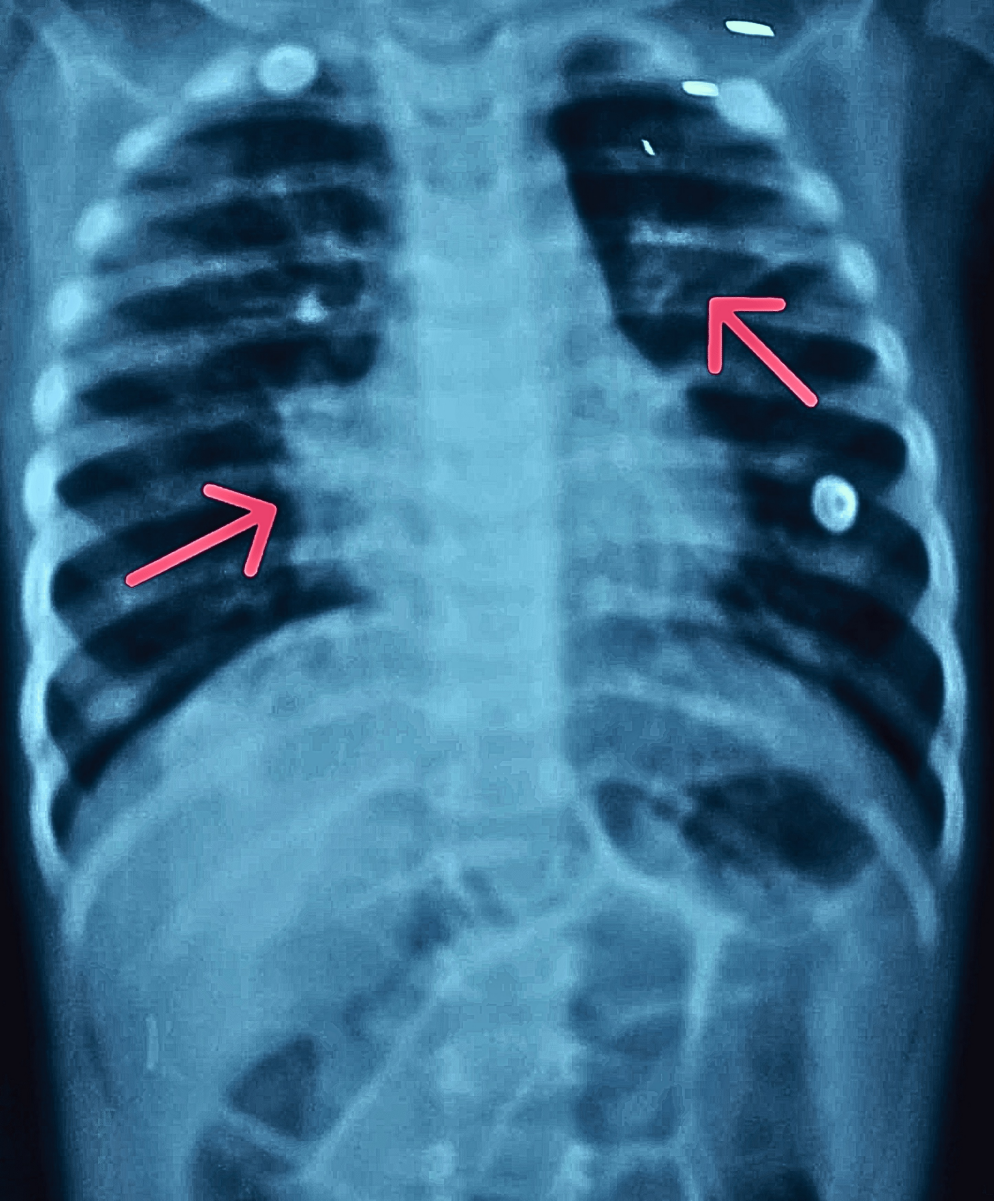
X-ray chest PA view showing consolidation in the right peri-hilar region and non-homogenous patchy opacities of the left middle lobe

The child subsequently developed stage 3 acute kidney injury (AKI) and underwent hemodialysis. Renal parameters normalized after a single session, with an improvement in urine output. Serial chest X-rays demonstrated gradual resolution of pneumonia (Figure 4).

The sepsis screen showed a declining trend. Shock resolved, and he was successfully extubated after 11 days of mechanical ventilation. After a total of 20 days of hospitalization, the child recovered clinically and was discharged in stable condition.

## Discussion

hMPV remains a significant cause of respiratory morbidity, particularly in vulnerable populations, such as young children, immunocompromised individuals, and the elderly. The cases presented here underscore the clinical complexity of hMPV co-infections, which are increasingly recognized as drivers of severe disease. Recent literature highlights that 6%-23% of hospitalized patients with respiratory infections exhibit hMPV co-infections, often exacerbating clinical outcomes such as hypoxemia, prolonged hospitalization, and ICU admissions [16-18]. Our cases align with these findings, demonstrating how adenovirus, SARS-CoV-2, and bacterial pathogens such as *A. baumannii *synergistically worsen hMPV-associated pneumonia, necessitating escalated care.

hMPV’s seasonal circulation in late winter and spring overlaps with other pathogens, such as RSV and influenza, complicating differential diagnosis and management [19,20]. The co-detection of hMPV with adenovirus (Case 1) and SARS-CoV-2 (Case 2) reflects the growing recognition of polymicrobial interactions in severe respiratory disease. Viral load dynamics and host immune responses likely modulate this severity, as high hMPV titers correlate with prolonged viral shedding and systemic inflammation [12,18]. For instance, Case 1’s persistent fever and dystonic complications may stem from hMPV-induced neuroinflammation, a rare but documented extrapulmonary manifestation. Similarly, Case 2’s rapid deterioration highlights how bacterial co-infections (*S. pneumoniae*) can amplify lung injury, necessitating broad-spectrum antibiotics despite the absence of bacterial growth in initial cultures.

The absence of targeted antivirals for hMPV compels reliance on supportive care, including oxygen therapy and mechanical ventilation, as seen in both cases [21]. While molecular diagnostics (e.g., BioFire® panels) enable rapid pathogen identification, their underuse in routine practice delays appropriate therapy. For example, Case 1’s initial antibiotic escalation (piperacillin-tazobactam to colistin) underscores the pitfalls of empirical treatment without virological confirmation, contributing to antimicrobial overuse [18,19]. Ribavirin and immunoglobulins have shown limited efficacy in severe hMPV cases, though evidence remains anecdotal [18]. Emerging therapies, such as fusion (F) protein inhibitors and monoclonal antibodies, are under investigation but not yet clinically available [13].

Immunocompromised states and comorbidities such as global developmental delay (Case 1) significantly heighten the risk of severe hMPV disease [14,20]. Prophylactic measures, including hand hygiene and masking, are critical in high-risk settings, as no vaccine exists [22,23]. Notably, maternally derived antibodies offer transient protection in infants, but waning immunity in older adults and immunocompromised individuals leaves these groups vulnerable to reinfection [24].

The pathogenesis of hMPV-related complications, such as optic atrophy (Case 1) or myocarditis, remains poorly understood. Recent studies suggest hMPV may trigger endothelial dysfunction or direct neuronal injury, warranting further mechanistic research [14,15]. Additionally, viral genotyping (e.g., subgroups A/B) could refine prognostic models, as genotype A is linked to higher pneumonia risk [20]. Global surveillance networks, as highlighted by WHO, are essential to track hMPV’s evolving epidemiology and inform public health strategies [19].

Beyond its well-documented respiratory manifestations, hMPV has been occasionally associated with extrapulmonary complications, including neurological features such as seizures and encephalopathy [24]. While the exact incidence is low, the pathophysiological mechanisms are thought to involve systemic inflammation and immune-mediated responses, which may be particularly consequential in vulnerable pediatric populations with pre-existing conditions, such as those highlighted in the present case series [25]. The absence of specific antiviral therapy for hMPV underscores that neurological management remains primarily supportive and symptom-directed. This highlights a critical gap in the therapeutic landscape, reinforcing the urgent need for continued research into targeted antiviral agents and preventive vaccines to mitigate the full spectrum of severe hMPV disease.

## Conclusions

hMPV co-infections pose complex clinical challenges amid overlapping respiratory pathogens. Cases highlight rapid diagnostics, prudent antimicrobial use, and tailored care. Current guidelines prioritize symptom management, but future antivirals and vaccines may reduce burdens on vulnerable populations.
